# ZIF-Derived Nitrogen-Doped Porous Carbons for Xe Adsorption and Separation

**DOI:** 10.1038/srep21295

**Published:** 2016-02-17

**Authors:** Shan Zhong, Qian Wang, Dapeng Cao

**Affiliations:** 1 State Key Lab of Organic-Inorganic Composites, Beijing University of Chemical Technology, Beijing 100029, P. R.China; 2 Institute of Nuclear Physics and Chemistry, China Academy of Engineering Physics, Mianyang 621900, People’s Republic of China

## Abstract

Currently, finding high capacity adsorbents with large selectivity to capture Xe is still a great challenge. In this work, nitrogen-doped porous carbons were prepared by programmable temperature carbonization of zeolitic imidazolate framework-8 (ZIF-8) and ZIF-8/xylitol composite precursors and the resultant samples are marked as Carbon-Z and Carbon-ZX, respectively. Further adsorption measurements indicate that ZIF-derived nitrogen-doped Carbon-ZX exhibits extremely high Xe capacity of 4.42 mmol g^−1^ at 298 K and 1 bar, which is higher than almost all other pristine MOFs such as CuBTC, Ni/DOBDC, MOF-5 and Al-MIL-53, and even more than three times of the matrix ZIF-8 at similar conditions. Moreover, Carbon-ZX also shows the highest Xe/N_2_ selectivity about ~120, which is much larger than all other reported MOFs. These remarkable features illustrate that ZIF-derived nitrogen-doped porous carbon is an excellent adsorbent for Xe adsorption and separation at room temperature.

Noble gas xenon plays an important role in human industrial development and has increasingly extensive applications in aspects of lighting^1^, medicine^2^,^3^, nuclear magnetic resonance^4^ and so on. In order to obtain pure Xe for further utilization, the energy-intensive cryogenic fractional distillation was often used to concentrate the noble gas Xe from air. Alternatively, researchers have also made great efforts to adopt various kinds of absorbents to study Xe adsorption^5^,^6^,^7^,^8^. However, Xe has a very low concentration (0.086 ppmv) in air, and the traditional adsorbents like zeolites hold only very low Xe uptake^9^, which leads to a great challenge for Xe adsorption and separation. Therefore, finding high capacity adsorbents with large selectivity to capture Xe (including adsorption and separation) is very important and significant.

Recently, metal-organic frameworks (MOFs) and covalent-organic polymers (COPs), as a new class of porous materials, have attracted enormous attention in the field of gas adsorption and separation owing to their tunable pore structures, modified functional groups, multidimensional networks and high porosity & BET surface areas etc^10^,^11^,^12^,^13^. Thallapally *et al*.^14^,^15^,^16^,^17^ carried out a series of experiments to explore Xe adsorption capacity on MOFs, and found that Ni/DOBDC with high surface area, uniform porosity and polarization of metal cations exhibits a large Xe uptake of 4.16 mol kg^−1^ at 1 bar and 298 K, which is higher than that of activated charcoal^17^. Besides, Greathouse *et al*.^5^,^18^ used computational methods to study Xe adsorption/separation from air on several kinds of MOFs, and explored the effects of pore size and framework topology on noble gas selectivity. Similarly, Snurr *et al*.^19^ performed grand canonical Monte Carlo (GCMC) simulations to predict Xe uptake and Xe/Kr separation, and further screened the promising MOFs for Xe/Kr adsorption and separation among 137000 hypothetical MOFs^20^. Moreover, some other groups^6^,^21^ also investigated Xe adsorption and separation on MOFs containing open metal sites. It is still a great challenge to search for this kind of absorbent with both high capacity and excellent selectivity.

Nevertheless, Ben *et al*.^22^,^23^ found that the fully carbonized PAF-1-450 shows not only high CO_2_ capacity of 4.5 mmol g^−1^ at 273 K and 1 bar, but also large selectivity of 209 for a 15/85 CO_2_/N_2_ mixture, which was greatly better than the matrix PAF-1, because the carbonization approach can achieve dehydrogenation of phenyl rings in PAF-1, and efficiently decrease the excluded effects of hydrogen atoms of phenyl rings on the gas molecules adsorbed. Interestingly, the recently reported MOF-derived nitrogen-doped porous carbons not only demonstrate excellent electrochemical performance as electrode materials^24^,^25^,^26^, but also exhibit the improved gas adsorption capacity for H_2_ and CO_2_^27^,^28^.

In this work, we adopted zeolitic imidazolate framework-8 (ZIF-8) and ZIF-8/xylitol composite as precursors to prepare nitrogen-doped porous carbons and further investigated the Xe adsorption and separation in the nitrogen-doped carbons. For comparison, we also explored the Xe adsorption and separation in the matrix ZIF-8. In addition, our previous GCMC simulations suggest that CuBTC among several representative MOFs and COFs shows the highest Xe uptake of 3.60 mmol g^−1^ at 1 bar and 298 K^29^. Therefore, we also experimentally synthesized the CuBTC material for Xe adsorption and separation^30^. On the one hand, we can confirm the rationality and accuracy of the GCMC prediction of Xe adsorption on CuBTC. On the other hand, we can demonstrate if the ZIF-derived nitrogen-doped porous carbon is better than CuBTC material for Xe adsorption or not. For discrimination, we marked the ZIF-8 derived nitrogen-doped carbon as Carbon-Z and the ZIF-8/xylitol composite derived nitrogen-doped carbon as Carbon-ZX. The detailed synthesis process was presented in elsewhere^24^,^26^,^30^ and also in Supporting Information.

## Results and Discussion

**Figure 1:**
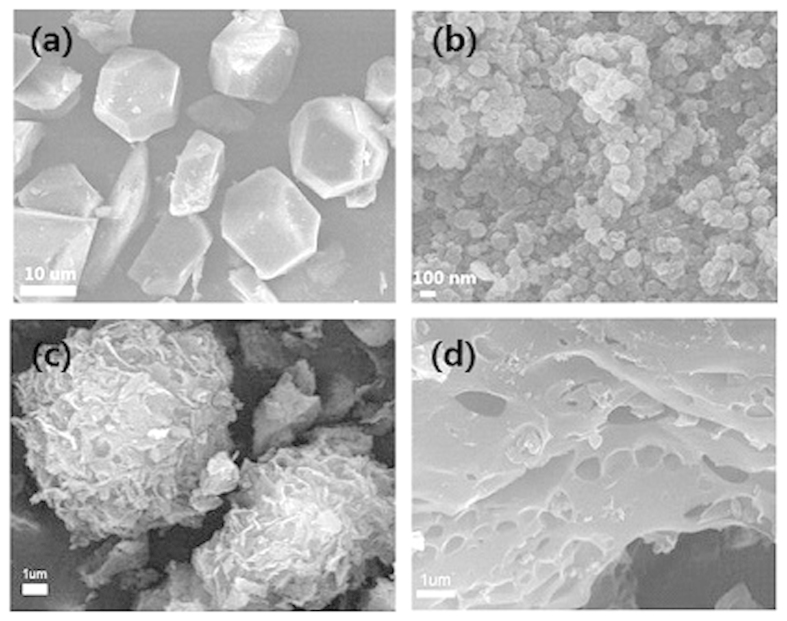
Scanning electron microscopy (SEM) images of (**a**) Cu-BTC, (**b**) ZIF-8, (**c**) Carbon-Z, and (**d**) Carbon-ZX.

Figure 1(a,b) displays the SEM images of Cu-BTC and ZIF-8 with their specific crystal appearance, which can be confirmed by the PXRD in Figure S1. It can be seen that the patterns agree well with the simulation peaks, meaning no impurity and a high purity phase. Figure 1(c,d) shows amorphous morphology of Carbon-Z and Carbon-ZX which coincides with their PXRD in Figure S2 with two typical peaks 2 θ ≈ 23° and 43° ascribed to the (002) and (101) planes of graphitic carbon^31^. Raman results in Figure S3 indicate the typical G band and D band, which originate from ideal sp^2^ carbons vibration in plane and defected carbons, respectively. Then, *I*_*G*_/*I*_*D*_ values of both Carbon-Z and Carbon-ZX are ~1.02, revealing the existence of graphitic and disordered structures in ZIF-derived porous carbons^32^,^33^. Besides, 2D peak also demonstrates the stack of graphite layer to some extent^33^,^34^,^35^. X-ray photoelectron spectroscopy (XPS) was also used to determine nitrogen content and found that the nitrogen contents of Carbon-Z and Carbon-ZX are 2.99% and 4.06%, respectively (Figure S4). Besides, Figure S4 also shows tthree different nitrogen types in Carbon-Z and Carbon-ZX, including pyridinic nitrogen, quaternary nitrogen and pyrrolic nitrogen^27^, implying the different bonding configurations in ZIF-derived nitrogen-doped porous carbons. Although there is no nitrogen element in xylitol, Carbon-ZX shows a higher nitrogen content than Carbon-Z, probably because the premelting and polymerization of xylitol molecules around ZIF-8 surface can protect the nitrogen losing in process of carbonization^26^.

**Figure 2:**
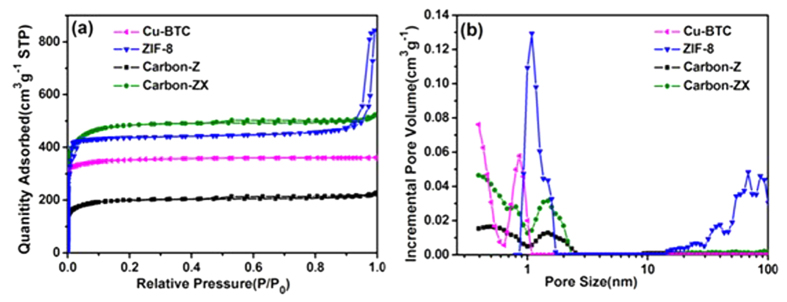
N_2_ adsorption-desorption and pore size distribution of Cu-BTC, ZIF-8, Carbon-Z and Carbon-ZX.

Adsorption–desorption isotherms of nitrogen at 77 K were used to further study the BET surface area and total pore volume by ASAP 2020 analyzer, and shown in Fig. 2. The porosity parameters are listed in Table 1. Obviously, Cu-BTC, Carbon-Z and Carbon-ZX exhibit type-I isotherm with almost all micropores (see Fig. 2b), while ZIF-8 displays type-IV isotherm with clear hysteresis hoop according to IUPAC classification^36^. Pore size distributions in Fig. 2 indicate that ZIF-8 possesses hierarchical pore structure including micropores less than 2 nm and meso- & macropores larger than 10 nm, while Cu-BTC only holds the micropore less than 1 nm, and both Carbon-Z and Carbon-ZX have micropores less than 2 nm. ZIF-8 holds the high surface area of 1321 m^2 ^g^−1^, while ZIF-8 derived Carbon-Z shows the smaller BET of 603 m^2 ^g^−1^, possibly owing to the partial collapse of skeleton in the process of heating. Surprisingly, compared with Carbon-Z, introducing additional carbon source ‘xylitol’ apparently improves the BET and pore volumes of Carbon-ZX (see Table 1). This possible reason is that in carbonization process, xylitol absorbed on ZIF-8 forms a premelting layer around ZIF-8 surface to keep skeletal integrity of product, which causes that Carbon-ZX possesses larger BET of 1470 m^2 ^g^−1^ and pore volume of 0.68 cm^3 ^g^−1^, compared to Carbon-Z. The observation is in good agreement with literature^25^,^37^.

**Figure 3:**
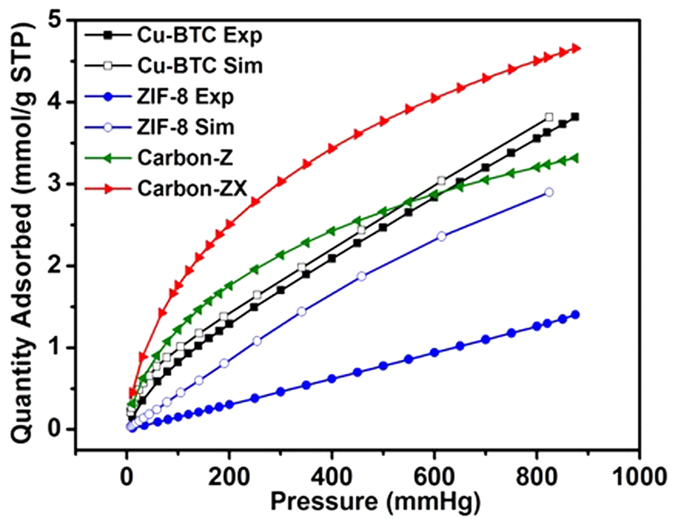
Experimental and simulated isotherms for Xe adsorption of Cu-BTC, ZIF-8, Carbon-Z and Carbon-ZX at 298 K, where simulation data is taken from Ref. 29.

Xe adsorption measurements at low pressure and 298 K were conducted using ASAP 2020 equipped with a constant temperature device by circulating water and glycol at 1:1 ratio, and the Xe adsorption isotherms are shown in Fig. 3, where all adsorption isotherms increase with pressure with no saturation. Interestingly, experimental uptake of Xe in Cu-BTC excellently matches with the GCMC simulation data^29^, suggesting that GCMC simulation is a powerful tool to predict the gas adsorption with high accuracy and rationality to some extent. However, Xe adsorption capacity of ZIF-8 was over-predicted in simulation way, which is largely higher than experimental results. This over-prediction phenomenon for gas capture on ZIF-8 also occurred in literature^5^, possibly owing to the crystal defects or marcopores existence of experimental product which are unfavorable for adsorption in practice. Compared to ZIF-8, Cu-BTC possesses higher Xe capacity of 3.39 mmol g^−1^ at 1 bar and 298 K, which is ascribed to open metal sites with polarity in Cu-BTC^16^,^20^.

It is noteworthy that Xe capacity of Carbon-ZX reaches 4.42 mmol g^−1^ at 1 bar and 298 K, which is a triple improvement compared to its matrix ZIF-8 (see in Table 1), although ZIF-8 and Carbon-ZX hold the similar BET surface area. This observation sufficiently indicates that the carbonization approach can efficiently achieve dehydrogenation of ZIF materials, and enhance the affinity of samples for Xe molecules by eliminating the excluded effects from hydrogen atoms of aromatic rings^23^,^28^. On the other hand, nitrogen-doping would lead to the asymmetrical distribution of charges on the carbon surface^38^,^39^, which also further enhances the electrostatic attraction between the charges on nitrogen-doped porous carbon surfaces and polarizable Xe molecules^22^,^40^. The synergistic effects of two reasons above result in the excellent performance of Carbon-ZX for Xe adsorption^22^,^41^.

Actually, Xe adsorption capacity of 4.42 mmol g^−1^ at 298 K and 1 bar for Carbon-ZX is not only higher than Cu-BTC, but also larger than almost all other pristine MOFs, including Ni/DOBDC of 4.16 mmol g^−1^, MOF-5 of 1.97 mmol g^−1^ under the same conditions^17^, monohalogenated IRMOF-2 series of 1.5–2.0 mmol g^−1^ at 292 K and 1 bar^42^, Al-MIL-53 of 2.0 mmol g^−1^ at 308 K and 1 bar^5^, CC3 of 2.69 mmol g^−1^ at 298 K and 1 bar^7^. However, Xe capacity of Carbon-ZX is slightly smaller than the value of 4.88 mmol g^−1^ for Ag-modified Ag@MOF-74Ni^15^. In short, ZIF-derived nitrogen-doped porous Carbon-ZX is an excellent candidate for Xe adsorption at low pressure and room temperature.

**Figure 4:**
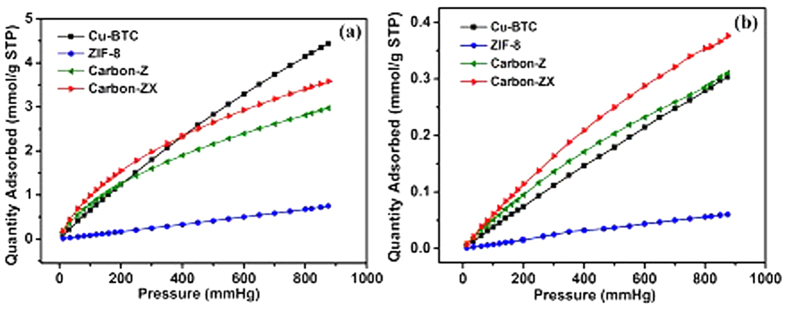
Experimental adsorption isotherms for gases on Cu-BTC, ZIF-8, Carbon-Z and Carbon-ZX samples at 298 K. (**a)** CO_2_, (**b**) N_2_.

**Figure 5:**
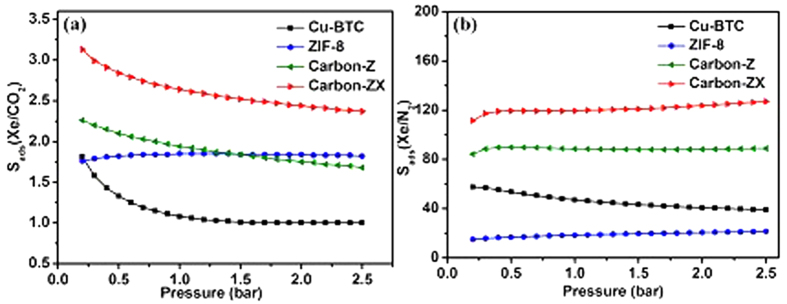
Adsorption selectivities of four samples for (**a**) Xe/CO_2_, (**b**) Xe/N_2_ at molar fractions *y*_CO2_ = 0.99, *y*_N2_ = 0.99, respectively.

Besides Xe adsorption, separation of Xe from mixtures is another important issue. Accordingly, we used ideal adsorption solution theory (IAST) as a credible tool to predict the gas selectivity^43^,^44^,^45^,^46^. Considering the fact that Xe has a very small concentration in air, therefore we plan to explore the selectivity for Xe/CO_2_ and Xe/N_2_ at the small ratio 1/99. Due to the requirement of IAST calculation, we also measured adsorption of CO_2_ and N_2_ adsorption in four samples at 298 K and their isotherms were shown in Fig. 4. The calculated selectivities for Xe/CO_2_ and Xe/N_2_ at the ratio of 1/99 were demonstrated in Fig. 5.

Figure 4 shows that ZIF-8 possesses the lowest adsorption for both CO_2_ and N_2_. Actually, the CO_2_ adsorption capacity of ZIF-8 at 1 bar coincides with the data reported in literature^47^. It is noted that Cu-BTC shows the highest CO_2_ adsorption at *p* > 0.5 bar compared with others, primarily attributed to the interactions between quadrupolar CO_2_ molecules and positive charges in unsaturated metal sites^48^. Furthermore, except Cu-BTC, both CO_2_ and N_2_ adsorption capacity follows the order of Carbon-ZX > Carbon-Z > ZIF-8, which means that ZIF-derived nitrogen-doped porous carbons hold remarkable property of gas adsorption.

Figure 5 shows the selectivity of four samples for Xe/CO_2_ and Xe/N_2_ at the same ratio 1/99. Clearly, Carbon-ZX exhibits the highest selectivity for both Xe/CO_2_ and Xe/N_2_ mixtures. With the increase of pressure, S_ads_(Xe/CO_2_) of Carbon-ZX gradually drops, while S_ads_(Xe/N_2_) almost keeps the same value about ~120.0 which is much higher than almost all other MOFs, for example, S = 11.7 ~ 32.0 of NOTT series, S = 5.3 ~ 18.2 of MOF-74 series, S = 44.0 of PCN-14^21^, and S < 10.0 of other MOFs including IRMOF-1 and UMCM-1^29^. Moreover, the selectivity trends for Xe/N_2_ follow the order of Carbon-ZX > Carbon-Z > CuBTC > ZIF-8 just like gas adsorption capacity ranking. Actually, Carbon-ZX possesses not only the highest Xe capacity but also the highest selectivities for both Xe/CO_2_ and Xe/N_2_ mixtures among four samples studied, which indicates that the ZIF-derived nitrogen-doped porous carbon is an excellent candidate in potential noble gas adsorption and separation from the air.

In summary, we have successfully synthesized nitrogen-doped porous carbons (Carbon-Z and Carbon-ZX) by using ZIF-8 and ZIF-8/xylitol composite as matrix. It is found that the prepared nitrogen-doped Carbon-ZX shows much better Xe adsorption properties, compared to matrix ZIF-8. Actually, the nitrogen-doped Carbon-ZX exhibits not only extremely high Xe adsorption capacity of 4.42 mmol g^−1^ at room temperature and 1 bar, which is larger than almost all other MOFs, but also excellent selectivity of ~120 for Xe/N_2_ at the same ratio 1/99, which is mainly attributed to the asymmetrical charge distribution on the nitrogen-doped porous carbon network and elimination of the excluded effects of H atoms on aromatic rings for the guests *via* carbonization. In previous reports, ZIF-derived nitrogen-doped porous carbons have shown excellent performance in supercapacitors and oxygen reduction reaction of fuel cells, while this work demonstrates that ZIF-derived nitrogen-doped carbon is also an excellent candidate for gas adsorption and separation, especially for noble gas Xe.

## Additional Information

**How to cite this article**: Zhong, S. *et al*. ZIF-Derived Nitrogen-Doped Porous Carbons for Xe Adsorption and Separation. *Sci. Rep*. **6**, 21295; doi: 10.1038/srep21295 (2016).

## Supplementary Material

Supplementary Information

## Figures and Tables

**Table 1:** BET surface area, total pore volume and Xe uptake at 298 K and 1 bar of four samples.

Sample	S_BET_(m^2 ^g^−1^)	V(cm^3 ^g^−1^)	Xe uptake(mmol g^−1^)
Cu-BTC	1090	0.46	3.39
ZIF-8	1321	1.17	1.21
Carbon-Z	603	0.30	3.17
Carbon-ZX	1470	0.68	4.42
