# Supplementary material for: ZIF-Derived Nitrogen-Doped Porous Carbons for Xe Adsorption and Separation
Source: Sci Rep. 2016 Feb 17;6:21295. doi: 10.1038/srep21295 (PMC4756705; doi:10.1038/srep21295)
Supplement: Supplementary Information [file srep21295-s1.doc]

ZIF-Derived Nitrogen-Doped Porous Carbons for Xe Adsorption and Separation

Shan Zhong ^1^, Qian Wang ^2^ and Dapeng Cao *^1^

^1^ State Key Lab of Organic-Inorganic Composites, Beijing University of Chemical Technology, Beijing 100029, P. R.China.

^2^ Institute of Nuclear Physics and Chemistry, China Academy of Engineering Physics, Mianyang 621900, People’s Republic of China

* Corresponding Author. Email: [caodp@mail.buct.edu.cn](mailto:caodp@mail.buct.edu.cn)

**S1. Experimental reagents**

The reagents involved in the synthetic courses are all analytical reagents (AR). Cupric nitrate trihydrate (Cu(NO_3_)_2_·3H_2_O), zinc nitrate hexahydrate (Zn (NO_3_)_2_·6H_2_O) were provided by Beijing Chemical Works. 2-methylimidazole and xylitol were purchased from Aladdin Chemical Co. Ltd. 1, 3, 5-benzene tricarboxylic acid were obtained from Alfa Aesar. Methanol and ethanol were provided by Sinopharm Chemical Reagent Co. Ltd. and Beijing Tongguang Fine Chemicals Company, respectively. The de-ionized water was made by Beijing University of Chemical Technology.

**S2. Synthesis of Cu-BTC**

Cu-BTC was prepared by hydrothermal method, as reported in literature.^1^ Firstly Cu(NO_3_)_2_·3H_2_O (1.75 g, 7.25 mmol) was dissolved in 37 mL de-ionized water in 50-mL beaker and 1, 3, 5-benzene tricarboxylic acid (1.05 g, 5 mmol) was dissolved in 37 mL ethanol in another 50-mL beaker. Then, the above solutions were mixed together in the hydrothermal reaction vessel. The sealed vessel was placed in the thermostatic oven, heated to 100 ^o^C and kept this temperature for 20 h, then cooled to room temperature. The reaction mixture was filtered and the product was washed with water and ethanol for three times. The final blue crystals were collected and evacuated at 80 ^o^C in vacuum for overnight.

**S3. Synthesis of ZIF-8**

For the synthesis of ZIF-8, as reported in literature,^2^ Zn(NO_3_)_2_·6H_2_O (0.735 g, 2.47 mmol) and 2-methylimidazole (H-MeIM) (1.62 g, 19.8 mmol) were added in 70 mL methanol in 100-mL round-bottom flask. The solution was stirred to dissolve completely. After standing for 1 h in the fume hood at room temperature, the mixture gradually became milky colloidal solution. Then, the mixture was transferred to centrifuge tubes. The product ZIF-8 was obtained by high-speed centrifugation at a speed of 8000 r min^-1^ for ~15 minutes and washed with methanol for several times, then followed by drying under vacuum at 70 ^o^C for 24 h.

**S4. Synthesis of ZIF-derived nitrogen-doped porous carbons ^3^**

The as-synthesized pure ZIF-8 was transferred into a quartz boat and placed in a furnace for carbonization. Before heating, argon gas flowed in the furnace for 2h for air out. The gas flow rate remained near 80 mL min^-1^ throughout the experimental process. The temperature was up to the 950 ^o^C at a rate of 3 ^o^C min^-1^, and maintained at T=950 ^o^C for 5 h under argon atmosphere followed by cooling down to the room temperature. The resultant product was denoted as Carbon-Z. For the synthesis of Carbon-ZX, the 0.5 g pure ZIF-8 was added in 20 mL 0.15 mol L^-1^ xylitol aqueous solutions in 50-mL beaker. Then, the beaker was placed on the magnetic stirrer with strong stirring at room temperature for 3 h. The resulting mixtures were stood overnight in the beaker, followed by filtration and washing with ethanol. Then, the ZIF-8/xylitol composite was also calcined in the similar procedures above. The resultant sample was labeled as Carbon-ZX.

**References**

1. Küsgens, P., et al. Characterization of metal-organic frameworks by water adsorption. *Micropor. Mesopor. Mater.* **120**, 325-330 (2009).

2. Zhang, L., et al. Highly graphitized nitrogen-doped porous carbon nanopolyhedra derived from ZIF-8 nanocrystals as efficient electrocatalysts for oxygen reduction reactions. *Nanoscale.* **6**, 6590-6602 (2014).

3. Zhong, S., Zhan, C., Cao, D. Zeolitic imidazolate framework-derived nitrogen-doped porous carbons as high performance supercapacitor electrode materials. *Carbon.* **85**,51-59 (2015).


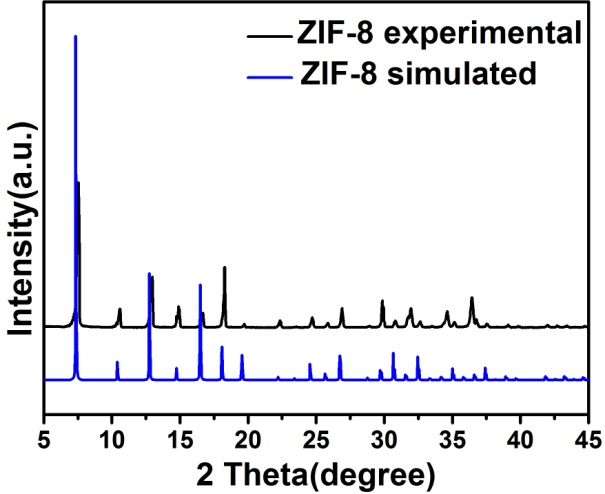

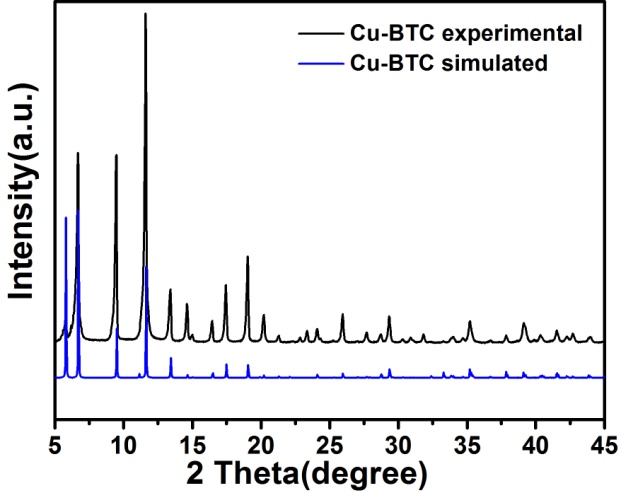


**Figure S1** Powder X-ray diffraction (XRD) patterns of Cu-BTC and ZIF-8


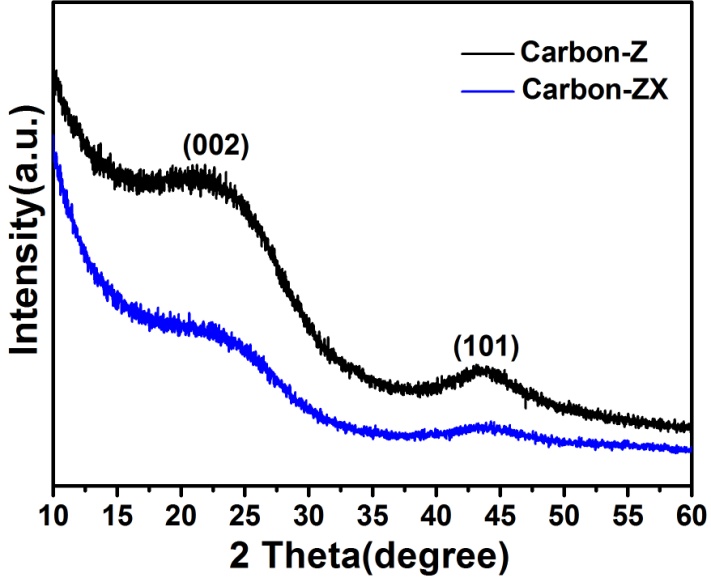


**Figure S2** Powder X-ray diffraction (XRD) patterns of Carbon-Z and Carbon-ZX


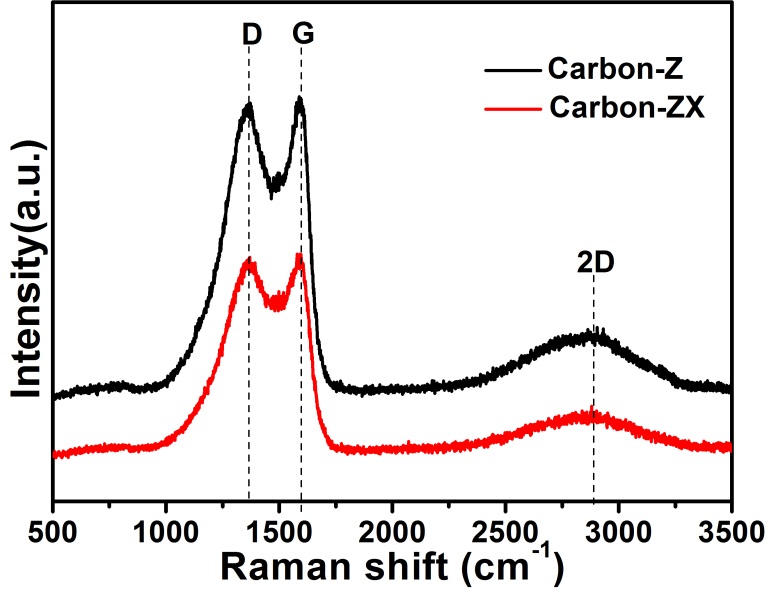


**Figure S3** Raman spectra of Carbon-Z and Carbon-ZX


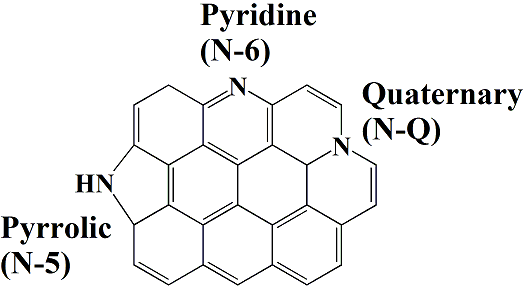


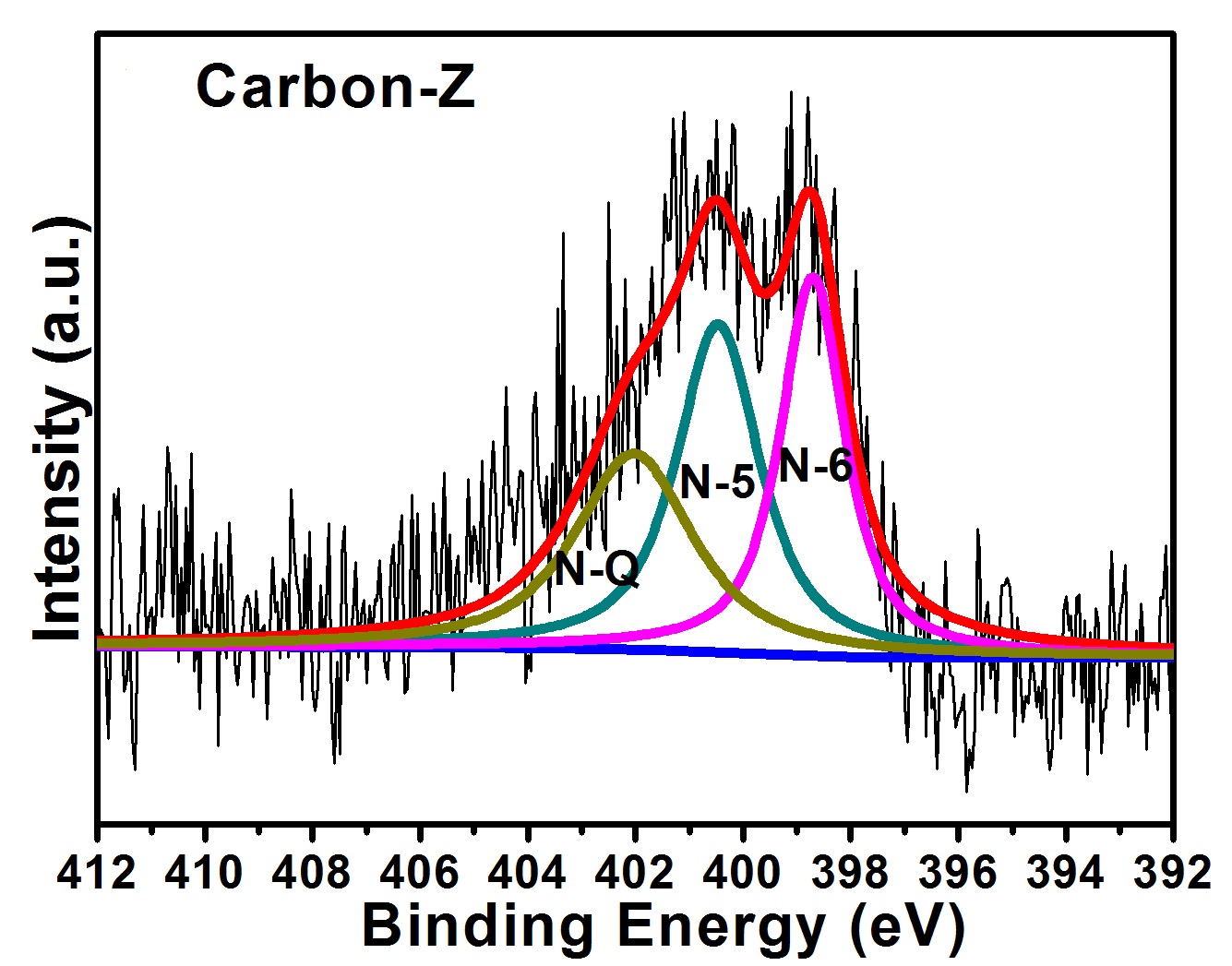

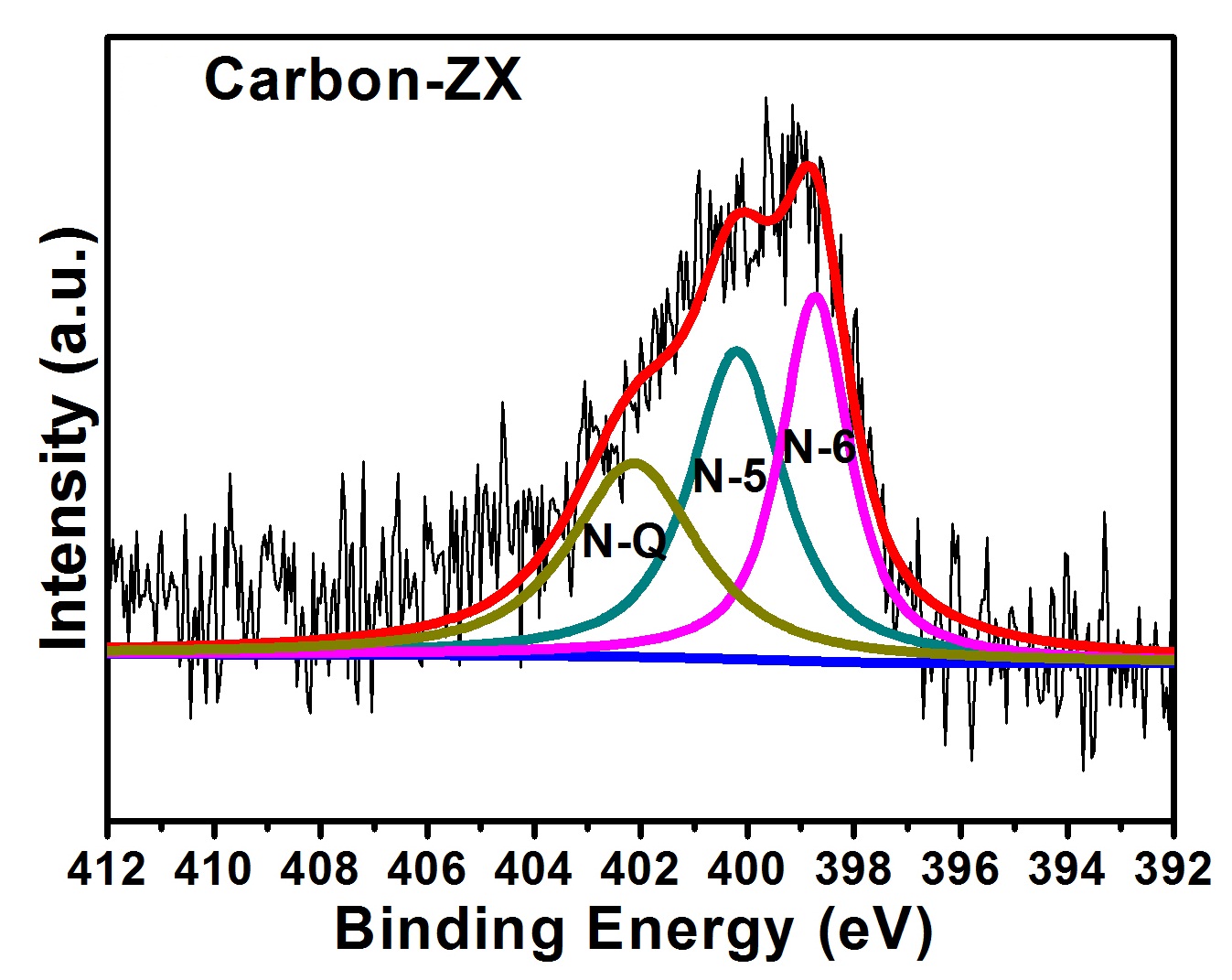


**Figure S4** Nitrogen contents and nitrogen types of Carbon-Z and Carbon-ZX
